# Corrosion Measurement of the Atmospheric Environment Using Galvanic Cell Sensors

**DOI:** 10.3390/s19020331

**Published:** 2019-01-15

**Authors:** Daiming Yang, Hongwei Mei, Liming Wang

**Affiliations:** Graduate School at Shenzhen, Tsinghua University, Shenzhen 518055, China; ydm15@mails.tsinghua.edu.cn (D.Y.); mei.hongwei@sz.tsinghua.edu.cn (H.M.)

**Keywords:** corrosion evaluation, atmospheric corrosion monitor (ACM), galvanic cell sensor, salt spray test

## Abstract

An atmospheric corrosion monitor (ACM) is an instrument used to track the corrosion status of materials. In this paper, a galvanic cell sensor with a simple structure, flexible parameters, and low cost was proposed for constructing a novel ACM, which consisted of three layers: the upper layer was gold, used as the cathode; the lower layer was corroded metal, used as the anode; and the middle layer was epoxy resin, used to separate the cathode and anode. Typically, the anode and epoxy resin were hollowed out, and the hollow parts were filled with electrolyte when it was wet to form a corrosive galvanic cell. Specifically, the corrosion rate was obtained by measuring the short circuit current of the cell. The sensor was made of a printed circuit board (PCB) or flexible printed circuit (FPC) and a metal coupon, which allowed for early control of the electrical parameters (including sensitivity and capacity) and could be combined with various metals. Additionally, the sensor feasibility was studied in water droplet experiments, during which the corrosive current changed with the electrolyte evaporation. The sensor practicability was also verified in a salt spray test, and the electric charge was compared using the thickness loss of bare coupons. A contrast test was also conducted for the corrosivity of different sensors made of aluminum, iron and copper.

## 1. Introduction

The assessment of the environment in which outdoor equipment with metal shells operate has attracted great interest in the fields of electronics, petroleum, and transportation [1,2,3]. However, metal equipment can easily be corroded in humid air, and the development of corrosion is reported to be related to the material species and the atmosphere [4,5].

Galvanic corrosion, a typical phenomenon that takes place in alloys or in the joints of different metals, is driven by the electrode’s potential difference between the corroded metal and the other conductors, including other metals or carbon [6]. Notably, the corroded metal is more active, with a low reversible potential and is used as the anode, while the other conductor serves as the cathode. Both are connected to each other to form a circuit loop, of which one part is the ion current in the electrolyte and the other part is the electron flow through the metal conductors. To be specific, a primary cell is constituted of two different metals or alloys covered by the electrolyte. Cast iron is a typical cell, in which iron and carbon act as the anode and cathode, respectively, when its surface is covered with a thin liquid film, thereby forming a tiny primary cell. The formed cells lead to iron corrosion, finally resulting in a rust-covered surface.

There are three typical methods to measure galvanic corrosion in the atmosphere: Coupon testing, electrochemical methods, and atmospheric corrosion monitors (ACM). Of these, a corrosion coupon is a simple and cost-effective tool used to quantitatively assess the corrosion rate of a particular metal in an outdoor environment. However, the exposure time may last for 1–20 years, due to the low rate of the atmospheric corrosion process [7]. Therefore, this test is time-consuming, and may only yield an annual corrosion rate [8]. Besides, it is impossible to obtain data during this process, such as the instantaneous rate in rain or fog. In contrast, other methods, including electrochemical impedance spectroscopy (EIS) [9,10,11], optical fiber [12,13], and microwave technology [14,15], are used to detect the corrosion status of metals. Nonetheless, these methods are not suitable for long-term continuous testing on site, due to their high requirements in terms of equipment. In comparison, ACM is a simple device that can monitor the galvanic corrosion rate of metals in the atmosphere in real time [16,17,18]. An ACM is structured with an open galvanic cell in case of wetting; the current follows through the cell and the anode metal is corroded. In this way, the corrosion rate can be tracked as a function of time using a single current sensor. Moreover, corrosivity is largely dependent on the thickness of the humidity layer and the pollutant deposition rate on the material surface.

In this study, traditional ACMs were first used for the corrosion tests. Using traditional ACMs, however, it was difficult to make the sensors and to maintain the consistency of the parameters. Moreover, they only allowed for continuous monitoring for days, rather than months. Therefore, this paper aimed to describe a new type of ACM sensor that was efficient in production and could easily regulate the sensitivity and capacity. In addition, the corrosivity was characterized through a water droplet experiment, and the practicability was also verified with the coupon testing results in the salt spray experiment.

## 2. Galvanic Cell Sensor

### 2.1. Galvanic Corrosion

When a metal (*M*) comes into contact with aqueous electrolyte solutions, an electrochemical reaction called corrosion occurs at the interface, which leads to deterioration or degradation of the metal. The metal is oxidized, which means that the metal loses electrons and becomes a metallic ion (*M^z^*^+^). Galvanic corrosion occurs between different metals connected through a circuit for current flow. This is driven by their potential difference [19]. Galvanic corrosion is also referred to as contact corrosion. The noble metal with more positive potential is not the reason for corrosion of the active metal with more negative potential, but for corrosion acceleration [20].

**Figure 1:**
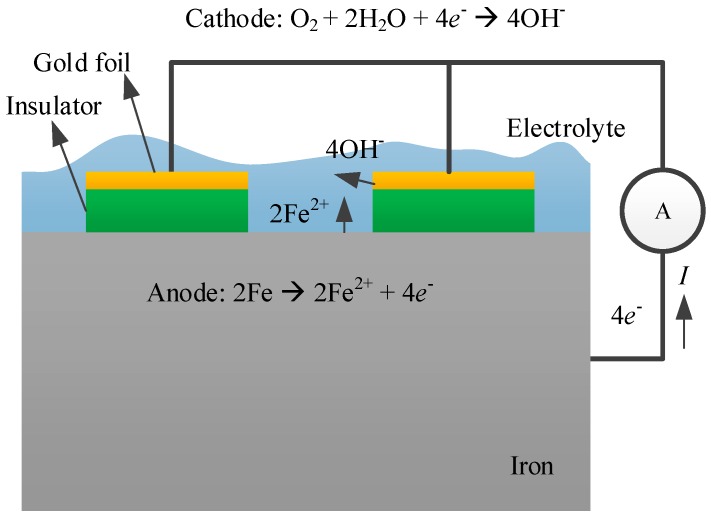
Schematic galvanic corrosion of a Fe-Au cell.

A galvanic cell, combining iron and gold foil, is shown in Figure 1. The two metals are connected by an external wire, and an ideal current meter without resistance is added to track the current. On the other side, the gold foil is insulated from the iron. If electrolytes cover the metals, a short circuit corrosion cell appears. The iron loses electrons and becomes a ferrous ion (Fe^2+^). These electrons pass through the current meter to the gold foil. Oxygen dissolved in the electrolyte obtains the electrons, combines with water, and becomes hydroxyl ions. According to Faraday’s laws of electrolysis, the mass of a substance dissolved or deposited during electrolysis is proportional to the quantity of electricity passed.

The mass loss (*m*) in micro grams (mg) is

(1) $$m=\frac{M}{nF}\cdot Q=\omega \cdot Q$$

where *M* is the molar mass of the metal in mg/mol; *n* is the valency number of ions of the metal; *F* is the Faraday constant with a value of 96500 C/mol; *Q* is the quantity of electricity or charge in C; and *ω* is the electrochemical equivalent. *Q* is accumulated by current (*I*) over time. The *ω* of iron (Fe-Fe^2+^) is 0.2893 mg/C. The equation ensures the availability of charge for the evaluation of corrosion.

### 2.2. Sensor Structure

**Figure 2:**
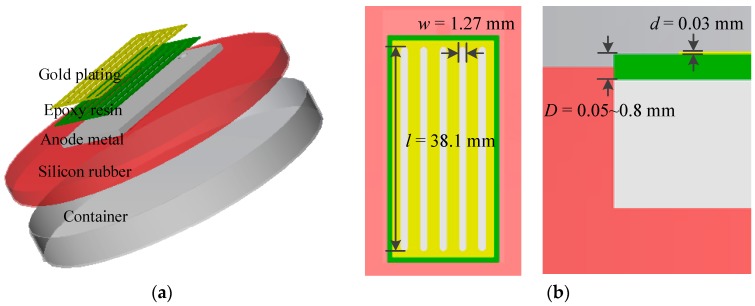
Component of a galvanic cell sensor (**a**) and its structure parameters (**b**).

Based on the galvanic corrosion principle above, we designed a corrosion sensor, namely a galvanic cell. The sensor is shown in Figure 2. The gold-plated epoxy resin plate, which was hollowed with five slots, was adhered to the surface of the corroded metal plate. The periphery of the epoxy board was filled with silicone rubber to prevent the external metal from being corroded. The entire sensor was placed in a plastic container, and the gold-plated layer was connected to the metal plate by a wire and a current meter.

To keep the thickness of the epoxy layer uniform and simplify processing, a printed circuit board (PCB) and flexible printed circuit (FPC) were applied to produce epoxy plates with gold-plated layers. Typically, the thickness of the epoxy layers of PCB is from 0.4 mm to 2.0 mm, and that of FPC is smaller, from about 0.05 mm to 0.2 mm. The literature showed that the insulating medium is from tens of microns to hundreds of microns in size [16,17,18]. The gold-plated layer was about 0.03 mm. The five slots were the same. The width was 1.27 mm and the length was 38.1 mm. They were parallel with an interval of 2.54 mm.

### 2.3. Key Parameters

**Figure 3:**
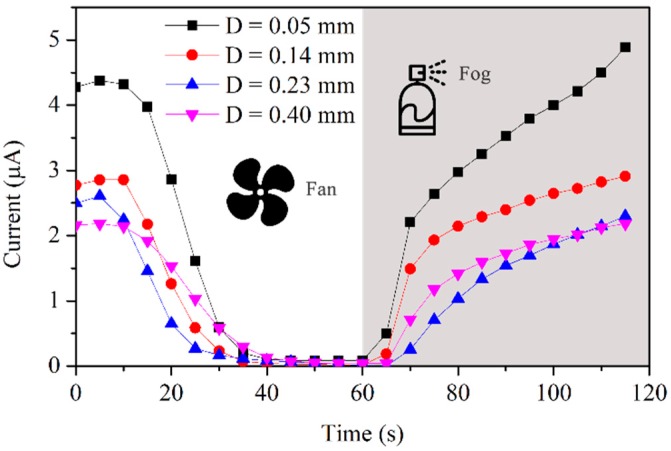
Sensor sensitivity described by the current versus the thickness of epoxy.

The key parameters of the sensor included sensitivity and capacity. Sensitivity is related to the thickness of the epoxy board and the gold-plated layer, especially the former. Corrosion will occur and the meter will acquire current only when the liquid film formed on the surface of the sensor links the gold-plated layer and the metal plate. A thicker epoxy board needs a thicker liquid film, and this results in a lower sensitivity of the sensor. Four sensors formed by epoxy plates with different thicknesses—0.05 mm 0.14 mm, 0.23 mm, and 0.40 mm—were used to test the sensitivity. They suffered from ultrasonic fog, and liquid films formed on the surface, so a fan was used to dry the surface. Wet and dry alternated in a cycle of 60 s. The changing current curves shown in Figure 3 displays the progress of corrosion. Large values of current result in a highly sensitive sensor. Therefore, the thinner sensor had higher sensitivity.

**Figure 4:**
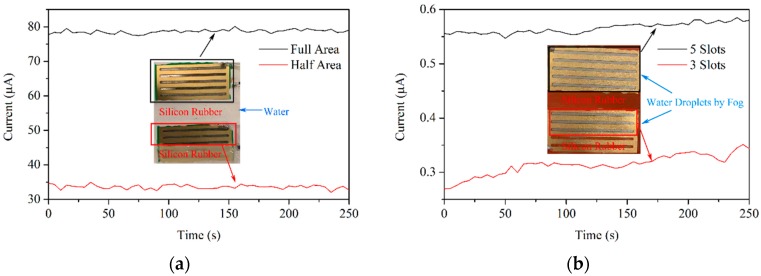
Sensor capacity described by the current versus the area of the gold-plated layer (**a**) and the number of slots (**b**).

The capacity is related to the area of the gold-plated layers and the perimeter of the slots. A large capacity means greater metal loss during corrosion, or that the electric charge and current is larger. Assuming that the sensor is completely wet and that the electrode reaction rate is determined by the oxygen diffusion rate, the capacity is proportional to the area of the gold-plated layer, as shown in Figure 4a. In humid air, where the liquid film is small and discrete, the capacity is proportional to the total circumference of the slots, as shown in Figure 4b. By the application of PCB and FPC design software, the area of the gold-plated layer and the circumference of the slots can be easily achieved.

Figure 4a shows two sensors—one of which has a full-area gold-plated layer and the other with a half-area silicon rubber layer—immersed in water, with steady-state corrosion currents of about 78 μA and 34 μA, respectively. The current ratio is 2.3 and close to that of the area. Figure 4b shows two other sensors—one of which has five slots and the other with three slots—that have been wet by ultrasonic fog. Tiny water droplets form on the surface, and the temporary steady-state currents are 0.55–0.58 μA and 0.27–0.35 μA, respectively. The ratio is close to that of the slot.

## 3. Water Droplet Experiment

### 3.1. Theoretical Calculation

Conductive water is dropped in the slots of the sensor, and the droplets are connected to the corroded metal and the gold-plated layer. Oxygen penetrates the droplets into the surface of the corroded metal or gold-plated layer, and a reduction reaction occurs. Electrons transferred from the corroded metal to the oxygen are reduced on the gold-plated layer, as determined by the current meter. Therefore, the galvanic corrosion rate can be obtained from the change of the current.

When the droplet is thick, the diffusion of oxygen in the solution is much slower than the reduction of oxygen [21], that is, the oxygen is consumed as soon as it reaches the surface of the gold-plated layer. The corrosion rate is limited by the oxygen diffusion.

Assuming that the diffusion of oxygen in solution satisfies Fick’s first law [22], the diffusion rate of the oxidant (*dm*_O_/*dt*) is defined as

(2) $$\frac{dm_{O}}{dt}=-D_{O}\cdot \frac{dc_{O}}{dx}$$

where the minus sign means that the diffusion direction is opposite to the concentration gradient (*dc*_O_/*dx*). *D*_O_ is the diffusion coefficient of the oxidant in the solution. The units of (*dm*_O_/*dt*), (*dc*_O_/*dx*) and *D*_O_ are mol/(cm^2^∙s), (mol/cm^3^)/cm, and cm^2^/s, respectively.

It is assumed that the diffusion of oxygen is in a constant state, that is, the diffusion speed at each point in the diffusion path is equal, and *D*_O_ is a constant. If the oxygen solubility of the solution in contact with the air is the oxygen saturation solubility, and the oxygen concentration of the solution in contact with the gold-plated layer is zero, the oxygen near the gold-plated layer is completely consumed. The consummation rate of the oxidant can be derived.

(3) $$\frac{dm_{O}}{dt}=-D_{O}\cdot \frac{c_{Oe}-c_{Os}}{l_{es}}$$

where *c*_O*e*_ and *c*_O*s*_ are the oxidant concentration at the interface of the electrode and the air, respectively. *l_es_* is the thickness of the droplet.

The corrosion current (*I*) can be described as follows:

(4) $$I=\frac{dn_{O}}{dt}\cdot z_{O}\cdot e$$

(5) $$\frac{dn_{O}}{dt}=\frac{dm_{O}}{dt}\cdot A\cdot N_{A}$$

where *z*_O_ is the electron loss of an oxidant molecule in the reduction reaction. *e* is electron charge, 1.60 × 10^−19^ C. (*dn*_O_/*dt*) is the oxidant consummation rate calculated as a molecule number. *A* is the area of the gold-plated layer covered by the solution. *N*_A_ is the Avogadro constant, 6.02 × 10^23^ C/mol.

### 3.2. Initial Corrosion

**Figure 5:**
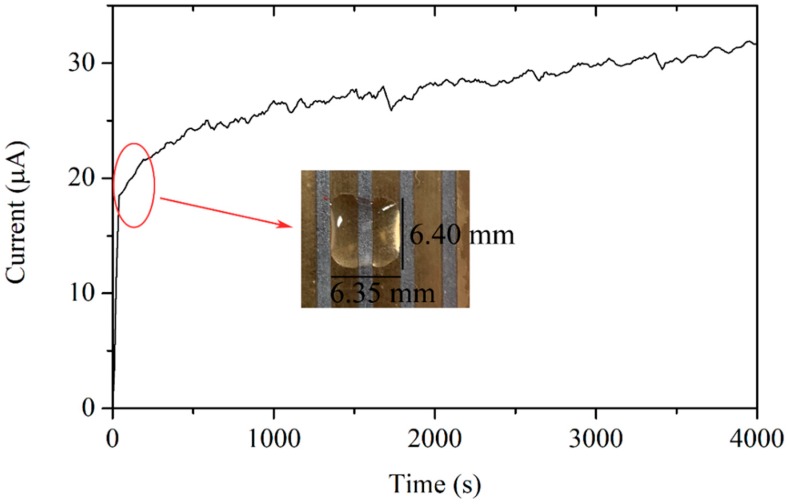
Salt water droplet on the sensor surface and the initial corrosion current.

To verify the calculation, a droplet of high-conductivity salt water of 20 μL was added to a corrosion sensor of *D* = 0.05 mm. The shape of the water droplet and the corrosion current curve are shown in Figure 5. Assuming that the water droplet was cuboid initially, the thickness of the solution and the area of the gold-plated layer covered by the solution can be obtained, and the parameters in Table 1 are substituted into (3)–(5) to calculate the theoretical maximum value of the initial corrosion current. The calculation value was 63 μA, on the same order of magnitude as the measurement in Figure 5.

The experimental curve shows that the initial corrosion current was about 20 μA, which was significantly lower than the theoretical maximum value. The causes of the difference include the following: the corrosion rate is not only related to the oxygen diffusion rate, but also affected by the cathode over potential; the discharge process of the charged particles through the electric double layer is not easy; and the reaction is affected by the discharge process.

### 3.3. Complete Process

**Figure 6:**
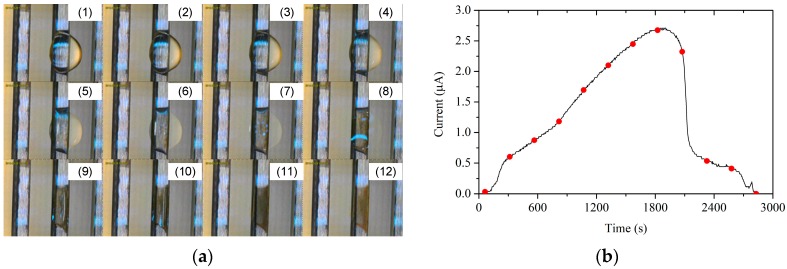
Change of a droplet on a sensor (**a**) and its current curve (**b**).

As shown in Figure 5, the corrosion current increases because of the evaporation of the droplet. To investigate the whole corrosion process, low-conductivity water was applied for the experiment. As time went on, the droplet thinned until it disappeared. Figure 6 illustrates the change of a droplet (5 μL) on a sensor of *D* = 0.4 mm, and the corresponding current curve. Photos were taken every 250 s, and the points when the photos were taken were marked on the curve. When corrosion began, the current increased as the thickness of the water thinned, when *l*_es_ decreased. When *A* reduced, the film on the gold-plated layer disappeared and the current dropped sharply, but not to zero. Finally, the current decreased slowly to zero.

**Figure 7:**
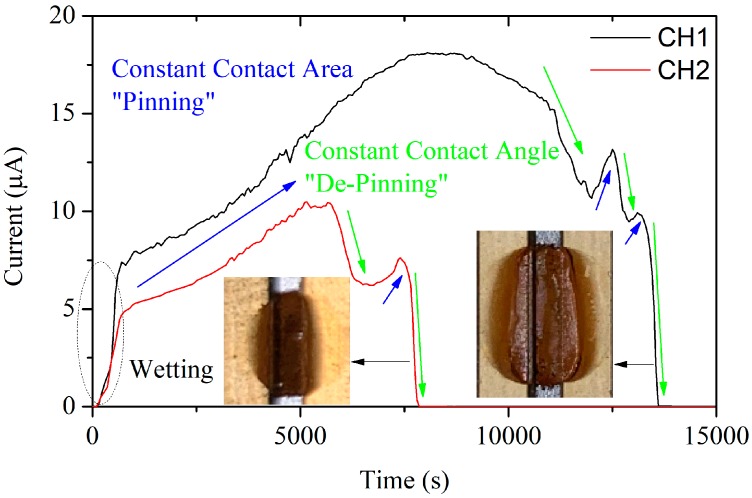
Comparison of two current curves under wetting by two wider droplets.

Wider droplets were applied for further tests. The current curves are displayed in Figure 7. Channel 1 was filled with a droplet about twice the size of that in channel 2. The larger droplet led to a larger maximum current, and its current lasted for a longer time. Compared with the curve in Figure 6, those in Figure 7 are more complicated. After the droplets wet the sensors, the currents increased slowly and synchronously to the maximum values. The evaporation of water made the film thin, but the wetting area on the gold-plated layer kept constant or changed little. This led to constant contact area evaporation, or “pinning” [23]. Then, the droplets shrunk, and the angle remained steady or even increased, that is, constant contact angle evaporation, or “de-pinning”. For a larger water droplet, the “pinning” and “de-pinning” steps would alternate until the droplet was dry. This phenomenon could be used for the detection of the wetting and corrosion status under wetting by droplets.

## 4. Salt Spray Test

### 4.1. Experiment Platform

**Figure 8:**
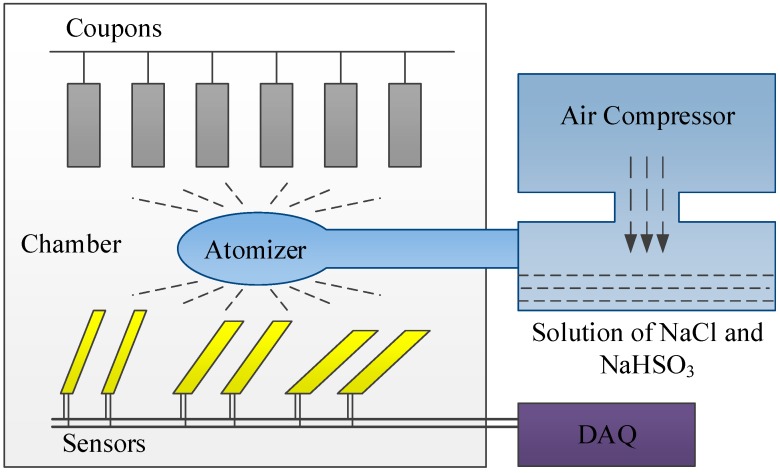
Platform and layout of the salt spray test.

The test was carried out in accordance with IOS 9227 [24]. The platform is shown in Figure 8. Three slopes were set in the salt spray box at 30°, 45°, and 60° from the horizontal plane, and two corrosion sensors of *D* = 0.4 mm were placed on each slope. Six pieces of iron coupons, which were the same material as the sensors, were suspended vertically in the box. The coupons were 50 mm × 25 mm × 2 mm. The solution contained NaCl and NaHSO_3_, simulating ocean and industrial environments. The temperature inside the chamber was 35 °C, and the temperature of the solution was 47 °C.

Salt fog was generated from the solution when an air compressor was subjected to high pressure. Fog filled the chamber uniformly via atomizers.

In the experiment, both the coupons and the sensors were corroded. The coupons lost iron and rust formed on the surface. The rust was cleaned using a rust remover, which was a kind of solvent that dissolved the rust but not the iron. Therefore, the weight loss of the coupons during the experiment could be obtained. The sensors were linked to a data acquisition (DAQ) system to track the corrosion currents and calculate the charges.

### 4.2. Charge–Thickness Loss

**Figure 9:**
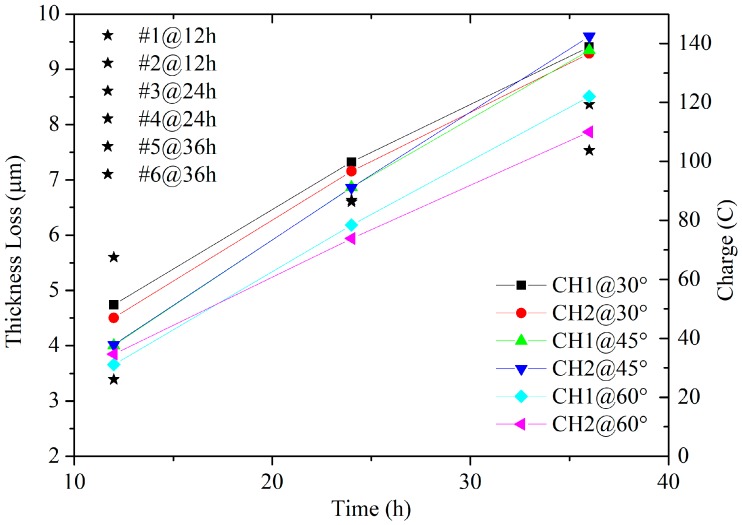
The relationship between the electric charges of the sensors and the thickness losses of the coupons in salt spray tests.

The coupons were taken out for weighing, and the weight loss was calculated. The experiment lasted about 36 h, and two coupons were weighed every 12 h. The weighed coupons were not put back into the chamber. The thickness loss of the coupons was calculated based on the weight loss, the surface area, and the density of the iron. The relationship between electric charges of the sensors and the thickness losses of the coupons is displayed in Figure 9.

In Figure 9, the thickness loss and the charge increased as the testing time progressed. The corrosion rates of the sensors at different angles were basically synchronized. A larger angle seemed to cause a slower rate, which might be due to a thicker film covering the sensor. In the early stage of the corrosion, the change of electric charge on the proposed sensor reflected a good linear growth, which also applied to the on-site detection of the atmospheric corrosion rate.

### 4.3. Material Types

Different metal materials were used to make the sensors, including aluminum (Al), iron (Fe), and copper (Cu). The corrosivity of the sensors based on these corroded metals was compared by a salt spray experiment.

According to the standard potential (*E*^0^) against the standard hydrogen electrode (SHE) for metal reduction, the *E*^0^ for aluminum, iron (Fe^2+^/Fe), and copper are −1.622 V, −0.440 V, and +0.337 V, respectively. The standard potential for oxygen (O_2_/OH^−^) is +0.401 V. Mathematically, the standard potentials for the galvanic cell sensors made by different metals are

(6) $$\mathrm{Al}-O_{2}:\;E_{cell}^{0}=E_{O_{2}}^{0}-E_{\mathrm{Al}}^{0}=2.023\;V$$

(7) $$\mathrm{Fe}-O_{2}:\;E_{cell}^{0}=E_{O_{2}}^{0}-E_{\mathrm{Fe}}^{0}=0.841\;V$$

(8) $$\mathrm{Cu}-O_{2}:\;E_{cell}^{0}=E_{O_{2}}^{0}-E_{\mathrm{Cu}}^{0}=0.064\;V$$

**Figure 10:**
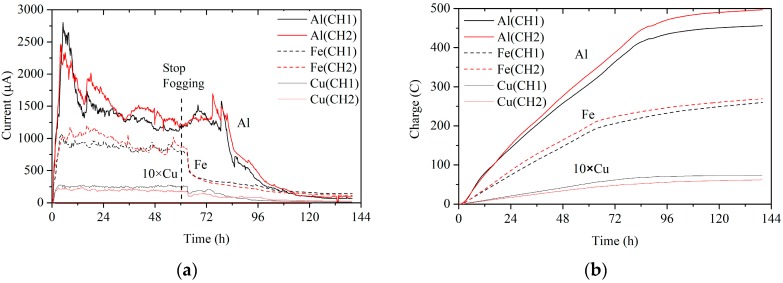
Corrosion currents (**a**) and electric charges (**b**) of sensors made of aluminum, iron, and copper in salt spray tests.

All of the cell potentials are positive, which means that corrosion will occur in aqueous solutions. Therefore, the metals could be corroded in salt spray experiments. The corrosion current and charge are illustrated in Figure 10.

The corrosion rates, expressed in current, were positively associated with the cell standard potentials. Among the three metals, aluminum was the most corrosive and copper was the least corrosive. The corrosion rate of copper was far behind that of the others. At about 60 h, the fog stopped being generated, but the sensors remained in the chamber for corrosion. As soon as the fog stopped, the corrosion of iron and copper slowed down. The aluminum, however, kept the same rate for about 20 h. It is well known that aluminum forms an oxide film or passivation film in the air to cover the surface and prevent further corrosion. At the beginning of the test, the formation of the oxide film suppressed the corrosion rate. The electrochemical reaction rate was mainly limited by the anodic reaction. Iron and copper did not form a passivation film, and during the fogging they were in sufficient contact with the electrolyte. The reaction rate was mainly limited by the diffusion of oxygen in the cathode. When the fogging stopped, the electrolyte on the surface of the sensor was lost, and the effective area of the galvanic cell was reduced. Meanwhile, the amount of oxygen participating in the cathodic reaction was reduced, and the corrosion rate decreased. The reduction of oxygen just after the fog stopping had little effect on the corrosion rate of aluminum.

The proposed sensor can be used to assess the corrosion rate of wet metal surfaces in the atmosphere. The rate is related to the form of wetting, including droplet and film, for which the sensor can measure the corrosivity. The sensor is easy to fabricate by simply attaching a gold-plated PCB or FPC to the surface of the corroded metal, and tracking the corrosion current through a current meter to estimate the corrosion rate. This is a generally applicable method. The sensor can be combined with an embedded DAQ module to create an atmospheric corrosion monitor for long-term, outdoor, or metal corrosion monitoring.

## 5. Conclusions

Based on the principle of galvanic corrosion, a new type of galvanic cell sensor was proposed. The sensors were fabricated using gold-plated PCB or FPC processes. The relationship between the thickness of the insulation layer and the sensitivity of the sensor was studied. The relationship between the area of the gold-plated layer, the length of the slots, and the capacity of the sensor was also studied.

Through the water droplet experiment, the corrosion rate was obtained during the evaporation of the droplet on the sensor surface. The corrosion rate was restricted by the diffusion of oxygen in the cathode. As the droplet evaporated and thinned, the corrosion current first increased, and then decreased. A droplet with a larger area led to a larger maximum corrosion current. The curve of the corrosion current reflected the pinning and de-pinning phenomena of the water evaporation process.

Through the salt spray test, the electric charge of the sensors and the thickness loss of the metal coupons were compared. In the early stage of corrosion, the sensor showed a good linear development trend, and was suitable for the on-site monitoring of metal corrosion.

Sensors made of different metals—aluminum, iron and copper—were compared in a salt spray test. The corrosion rates of the three metals were found to be related to the standard potentials of the cells. The larger the electrode potential, the faster the corrosion rate. At the same time, the passivation film suppressed the corrosion by slowing down the anodic reaction. Therefore, the sensors made of different metals exhibited different corrosion trajectories in the test.

## Figures and Tables

**Table 1:** Parameters for calculation of the maximum initial current.

Parameter	Value	Unit
*D*_O_	1.9 × 10^−5^	cm^2^/s
*c*_Oe_	0	mol/cm^3^
*c*_Os_	1.38 × 10^−6^	mol/cm^3^
*l*_es_	5 × 10^−2^	cm
*z*_O_	4	-
*A*	3.2 × 10^−1^	cm^2^

## References

[1] Hamidi R.J., Hosseinian S.H., Sadeghi S.H., Qu H.Z. (2015). A Novel Approach to Utilize PLC to Detect Corroded and Eroded Segments of Power Transmission Lines. IEEE Trans. Power Deliv..

[2] Yuan X., Li W., Chen G., Yin X., Yang W., Ge J. (2018). Two-Step Interpolation Algorithm for Measurement of Longitudinal Cracks on Pipe Strings Using Circumferential Current Field Testing System. IEEE Trans. Ind. Inform..

[3] Chung H., Yang C., Jeung G., Jeon J., Kim D. (2011). Accurate Prediction of Unknown Corrosion Currents Distributed on the Hull of a Naval Ship Utilizing Material Sensitivity Analysis. IEEE Trans. Magn..

[4] Bardal E. (2004). Wet Corrosion: Characteristics, Prevention and Corrosion Rate. Corrosion and Protection.

[5] Chico B., de la Fuente D., Díaz I., Simancas J., Morcillo M. (2017). Annual Atmospheric Corrosion of Carbon Steel Worldwide. An Integration of ISOCORRAG, ICP/UNECE and MICAT Databases. Materials.

[6] Cottis B., Graham M., Lindsay R., Lyon S., Richardson T., Scantlebury D., Stott H. (2010). Environmental Modification for Cooling, Heating and Potable Water Systems. Shreir’s Corrosion.

[7] ISO (2011). Metals and Alloys—Atmospheric Corrosion Testing—General Requirements.

[8] Morcillo M., Chico B., Díaz I., Cano H., de la Fuente D. (2013). Atmospheric corrosion data of weathering steels. A review. Corros. Sci..

[9] Du C., Owusu Twumasi J., Tang Q., Guo X., Zhou J., Yu T., Wang X. (2018). All-Optical Photoacoustic Sensors for Steel Rebar Corrosion Monitoring. Sensors.

[10] Ma C., Song S., Gao Z., Wang J., Hu W., Behnamian Y., Xia D.H. (2017). Electrochemical noise monitoring of the atmospheric corrosion of steels: Identifying corrosion form using wavelet analysis. Corros. Eng. Sci. Technol..

[11] Fu X., Dong J., Han E., Ke W. (2009). A New Experimental Method for in Situ Corrosion Monitoring Under Alternate Wet-Dry Conditions. Sensors.

[12] Wei H., Zhao X., Kong X., Zhang P., Cui Y., Sun C. (2014). The Performance Analysis of Distributed Brillouin Corrosion Sensors for Steel Reinforced Concrete Structures. Sensors.

[13] Zhao X., Gong P., Qiao G., Lu J., Lv X., Ou J. (2011). Brillouin Corrosion Expansion Sensors for Steel Reinforced Concrete Structures Using a Fiber Optic Coil Winding Method. Sensors.

[14] Zhang H., He Y., Gao B., Tian G.Y., Xu L., Wu R. (2016). Evaluation of Atmospheric Corrosion on Coated Steel Using *K*-Band Sweep Frequency Microwave Imaging. IEEE Sens. J..

[15] Sutthaweekul R., Tian G.Y. (2018). Steel Corrosion Stages Characterization Using Open-Ended Rectangular Waveguide Probe. IEEE Sens. J..

[16] Mizuno D., Suzuki S., Fujita S., Hara N. (2014). Corrosion monitoring and materials selection for automotive environments by using Atmospheric Corrosion Monitor (ACM) sensor. Corros. Sci..

[17] Shi Y., Fu D., Zhou X., Yang T., Zhi Y., Pei Z., Zhang D., Shao L. (2018). Data mining to online galvanic current of zinc/copper Internet atmospheric corrosion monitor. Corros. Sci..

[18] Cao X., Deng H., Lan W., Cao P. (2012). Electrochemical investigation on atmospheric corrosion of carbon steel under different environmental parameters. Anti-Corros. Methods Mater..

[19] Perez N. (2016). Electrochemical Corrosion. Electrochemistry and Corrosion Science.

[20] Cao C. (2008). Non-Equilibrium Potential. Principles of Electrochemistry of Corrosion.

[21] Tsuru T., Nishikata A., Wang J. (1995). Electrochemical Studies on Corrosion Under a Water Film. Mater. Sci. Eng. A.

[22] Perez N. (2016). Mass Transport by Diffusion and Migration. Electrochemistry and Corrosion Science.

[23] Anantharaju N., Panchagnula M., Neti S. (2009). Evaporating drops on patterned surfaces: Transition from pinned to moving triple line. J. Colloid Interface Sci..

[24] ISO (2017). Corrosion Tests in Artificial Atmosphere—Salt Spray Tests.

